# Collaboration Expertise in Medicine - No Evidence for Cross-Domain Application from a Memory Retrieval Study

**DOI:** 10.1371/journal.pone.0148754

**Published:** 2016-02-11

**Authors:** Jan Kiesewetter, Frank Fischer, Martin R. Fischer

**Affiliations:** 1 Institut für Didaktik und Ausbildungsforschung in der Medizin, Klinikum der Ludwig-Maximilians-Universität München, München, Germany; 2 Department of Educational Science and Educational Psychology, Ludwig-Maximilians-Universität München, München, Germany; University of Groningen, NETHERLANDS

## Abstract

**Background:**

Is there evidence for expertise on collaboration and, if so, is there evidence for cross-domain application? Recall of stimuli was used to measure so-called internal collaboration scripts of novices and experts in two studies. Internal collaboration scripts refer to an individual’s knowledge about how to interact with others in a social situation.

**Method—Study 1:**

Ten collaboration experts and ten novices of the content domain social science were presented with four pictures of people involved in collaborative activities. The recall texts were coded, distinguishing between superficial and collaboration script information.

**Results—Study 1:**

Experts recalled significantly more collaboration script information (*M* = 25.20; *SD* = 5.88) than did novices (*M* = 13.80; *SD* = 4.47). Differences in superficial information were not found.

**Study 2:**

Study 2 tested whether the differences found in Study 1 could be replicated. Furthermore, the cross-domain application of internal collaboration scripts was explored.

**Method—Study 2:**

Twenty collaboration experts and 20 novices of the content domain medicine were presented with four pictures and four videos of their content domain and a video and picture of another content domain. All stimuli showed collaborative activities typical for the respective content domains.

**Results—Study 2:**

As in Study 1, experts recalled significantly more collaboration script information of their content domain (*M* = 71.65; *SD* = 33.23) than did novices (*M* = 54.25; *SD* = 15.01). For the novices, no differences were found for the superficial information nor for the retrieval of collaboration script information recalled after the other content domain stimuli.

**Discussion:**

There is evidence for expertise on collaboration in memory tasks. The results show that experts hold substantially more collaboration script information than did novices. Furthermore, the differences between collaboration novices and collaboration experts occurred only in their own content domain, indicating that internal collaboration scripts are not easily stored and retrieved in memory tasks other than in the own content domain.

## Introduction

A chief of medicine joins a group of physicians and nurses in a ward-round. The group stands at a patient’s bed while one of the physicians repeats the information gathered on the patient thus far. At this point, none of the other physicians have successfully diagnosed the patient. The group therefore discusses two different ideas on how to rule out one or the other diagnosis. The chief of medicine has been standing mute in the background. He clears his throat and, as the group turns around to him, starts asking questions about the two possible examination techniques and their value toward different diagnoses. None of the team members seems surprised by the questions because the chief of medicine is known to ask questions when participating in a ward-round. All questions are answered by different members of the group, who themselves start to ask questions of one another. After some time has passed, two team members almost simultaneously shout out that one diagnosis is much more probable than the other, to which the chief of medicine nods and the group continues organizing issues regarding the patient’s further diagnostic examinations. Later in the day, the chief of medicine attends an apartment owners meeting at which four of the other apartment owners are discussing two different strategies regarding retail investments in the next quarter.

This example models important questions: First, despite the physicians finally coming up with the conclusion, why did the chief of medicine know the diagnosis right away but not share it? Second, why did the chief of medicine not simply tell the group the diagnosis, but rather ask the group so that they generated the solution themselves? Third, will the chief of medicine be able to deploy a similar tactic in solving the problem in his apartment owners meeting? The questions are relatable to expertise research beyond medical or diagnostic expertise. The chief of medicine seems to have expert medical knowledge about the patient. More than that, however, he seems to have embedded collaborative activities into the working environment. Furthermore, he appears to have expertise in this collaborative situation at the patient’s bed, as he started to ask questions, which helped the group to overcome a point of disagreement. In the comparable situation at his apartment owners meeting, it is unclear whether the chief of medicine will think to deploy the same collaborative activities or even whether they could be helpful.

This article explores in two empirical studies whether it is possible to find expertise on collaboration and if so, whether the collaboration expertise can applied across domains. Answering these questions is a precondition for transferring the results into educational practice and shaping tailored educational courses on collaboration.

Collaboration expertise has rarely been approached directly in the literature, so the following sections concentrate on three lines of research to explain and contrast collaboration expertise to other expertise domains. We recapitulate research on expertise and collaboration scripts to formulate hypotheses comparing how experts and novices might behave when confronted with a collaborative situation.

### Expertise in Collaboration

According to Gruber and Mandl [1], an expertise domain is limited in its content, tasks, and actions and carries a specific but limited set of knowledge. One could ask why expertise in collaboration has not been studied. Two reasons are possible: First, performance in collaboration often is strongly covariant with a specific task (i.e., the score of a basketball team reflects collaborative activity as well as the individual ability to throw baskets) [2]. Further, this task, due to its specificity, is in its nature dynamic and difficult to standardize and reproduce and therefore difficult to assess [3]. The second difference between expertise in collaboration and other expertise domains that have been studied (such as chess or sports) is that the former requires a second domain—the so-called content domain, in which experts collaborate. Expert-novice comparisons are thus more difficult to conduct as there are four groups which in principle can be compared: experts in both, collaboration and the content domain, and experts in only collaboration, only the content domain and novices in both, collaboration and the content domain. In practice the collaborative expertise of participants is built up in parallel to expertise in the content domain. The result for an assessment is that performance indicators of collaborative activities are naturally a result from the proficiency of not one, but actually two interwoven tasks. On the one hand there is the ability to master this exact collaborative situation (a certain level of knowledge and skills is necessary to do so) and on the other hand there is the individual ability to master the task of the content domain woven into this collaborative situation (again, with a certain level of knowledge and skills necessary to do so). Methodologically tasks have two difficulties, two reliabilities, two objectivities. In practice, the assessor only has one performance indicator for collaborative expertise inseparable from performance in the content domain.

The nature of the interaction between the two expertise domains—namely the domain of collaboration and the content domain—has not been investigated thus far. For example, whether expertise in collaboration is transferable to other content domains is unclear. Studies focusing on the transferability of general cognitive skills concluded that expert performance is limited in its scope, and thus expert performance does not transfer [4]. However, these studies were conducted in a single domain. One study found that, through the so-called “tool” domain of reading, learning new domain-specific words and concepts can be acquired in various content domains. The authors of that study conclude that transferability could, to a certain extent, be found [5]. Whether expertise in collaboration does exist and is measurable and whether it is dependent on the content domain have not been studied so far.

Despite the differences between the diverse content domains, how experts use their knowledge has many common attributes. Research, has put great effort in investigating and capturing these attributes. The following sections review this literature and discuss how the attributes of expertise might apply to a possible expertise domain of collaboration.

### Attributes of Expertise

Expertise in many domains has been subject to research from various directions. Whereas performance is measurable and reliably distinguishes experts from novices, how experts use their knowledge is much more difficult to assess. Among the “classic” domains of expertise research (e.g., chess, sports, music), some similarities can be identified. Experts create more effective solutions for problems in their domain; recognize the structure underlying problems of their domain; possess extensive memory for information in their domain; and have deeper, better structured knowledge [6–8]. These features are presented next and illustrated by selected empirical research results.

Experts do not necessarily consider more *possible* solutions to a problem; rather, they find more *effective* ones, as they try to understand problems before applying solutions too quickly (cf. [9]). When doing so, experts are more effective than novices in recognizing the general structure underlying problems in their domain and setting aside surface features of the problems [10]. Early theorists favored the idea of general cognitive skills (applicable to a broad variety of problems [11]) to explain expert performance. Later, local knowledge was identified as the key determinant of expert performance [12]. This specialized knowledge was found to be highly structured around typical configurations [13].

This highly structured knowledge would currently be explained by an advancement of the expert’s script with a superior script recall. In one investigation, Custers, Boshuizen, and Schmidt [14] approached doctors with varying expertise, gave the name of a disease, and asked them to write down the prototypic patient and clinical pattern. The more experience the physician had, the more detailed the description. Experts actively wrote down patient information without being asked to, whereas novices activated this knowledge only if instructed to do so. The experts seem to have built configurations of disease-relevant information with additional knowledge about patients through their experiences (for a summary on expertise and experience, see [1]). This prototypic knowledge was referred to as “illness scripts” by Custers et al. [14]. In their view, expertise was revealed through a qualitative shift of first accumulating causal knowledge, then encapsulating this knowledge into high-level but simplified causal models, and finally using illness scripts in treating novel patient problems.

The concept of illness scripts builds on the idea of Schank and Abelson [15] in that knowledge of the same kind is clustered together. In these authors’ theoretic view, scripts in general are defined as “culturally shared knowledge about the world that provides information about conditions, processes, and consequences of particular everyday situations.” They describe scripts for diverse situations, such as visits to restaurants or the dentist, and state that, through repeated experience, procedural knowledge is stored in scripts. Explicating this idea, knowledge of a particular area, known as an internal script, is sorted into established structures of memory [16]. Such internal scripts are assumed to be acquired through repeated experiences in a certain class of situations [17]. The idea that experiences change the way people perceive situations has also been studied in research regarding “professional vision” in the field of teacher education [18].

If there is indeed evidence for collaboration as an expertise domain, the following should apply: (1) The collaboration experts should create more effective solutions than novices for problems in collaborative situations. The solutions created by collaboration experts should employ “conditionalized” knowledge, which can be deployed fluently or even automatically in a collaborative situation. (2) Collaboration experts should be effective at recognizing the structure underlying collaborative problems. Whereas novices might focus more on superficial features of a collaborative task, experts should be able to understand the basic structure of a collaborative situation. (3) Collaboration experts should possess extensive memory for collaborative information. As with other expertise domains, they should have a greater amount of collaboration information stored in memory. (4) Collaboration experts should have a deeper and better structured knowledge on collaboration. They should be able to organize collaborative information and should consider patterns of collaborative situations. The latter aspect, the structuring of knowledge, has been investigated through the script concept in expertise research as well.

### Collaboration scripts as components of collaboration expertise

If collaboration expertise exists, collaboration knowledge of experts should be distinguishable from that of novices. The script concept has been useful also in identifying differences in other expertise domains (e.g., [14]). This research includes the concept of illness scripts as hierarchically organized knowledge structures; such collaboration scripts include knowledge about where collaboration is derived and how it might develop. Early script approaches modeled scripts to be of linear logic—once started they would be processed in the one and only order until the end of the script. Besides application in artificial intelligence programming, the scripts were difficult to adapt to the real world [19]. However, in the past decade, the instructional power of scripts has been revealed through their function in computer-supported collaborative situations [17].

Collaboration scripts are knowledge structures, developing over time in collaborative situations. Two kinds of collaboration scripts have been distinguished in the literature: internal and external collaboration scripts. When learners engage in high-level collaborative activities, external collaboration scripts are textual or graphical representations of a collaborative practice, which guide learners. “[External] Collaboration scripts provide more or less explicit and detailed instructions for small groups of learners on *what* activities need to be executed, *when* they need to be executed, and by *whom* they need to be executed in order to foster individual knowledge acquisition” [20]. Examples of external collaboration scripts are explanations or questions embedded in a computer-supported collaborative learning environment.

External collaboration scripts have been realized without computers as well. Examples for learning new content from a textbook would be that learning partner A summarizes a paragraph for learning partner B, and then learning partner B adds some paragraph to the summary, and finally learning partner A criticizes the addition to the summary. When learners have spent several learning sessions in such an environment, these collaborative activities are thought to be internalized, thus forming internal collaboration scripts. Theoretically, internal collaboration scripts are thought to be largely parallel in structure to external collaboration scripts. Empirical results have shown that external collaboration scripts are effective in advancing computer-supported [21–23] and face-to-face collaborative activities (e.g., [5,24]).

Fischer et al. [17] have provided a theoretical outline of collaboration scripts in the Script Theory of Guidance (SToG). In their theory, these authors build upon two basic theories: First is the theory of dynamic memory, in which internal scripts are sought to be adaptable in situations and change due to experience. Second, they built upon sociocultural perspectives, incorporating the idea of a zone of proximal development by Vygotskiĭ [25], in particular as the difference between what a learner can do without help and what he or she can do with help. The SToG is an extensive work (considering four components and seven principles); here, we focus on the four SToG structural components of collaboration scripts (plays, scenes, scriptlets, and roles) that guide the understanding of the collaboration.

The *play* is the knowledge about the participants’ situation (e.g., an argumentative dialogue). The play component is built hierarchically, consisting of multiple scenes and knowledge about the sequence of these scenes being incorporated within the play. A *scene* then includes knowledge about situations within the play (e.g., a counter-position is part of an argumentative dialogue). A scene consists of several *scriptlets* that incorporate knowledge of sequences of activities within the scene (e.g., stating a counter-position before presenting the evidence for it). The *roles* component incorporates the knowledge of participants in a collaborative situation. The roles define which behavior is and is not adequate for each role (e.g., one participant might represent the pro arguments and the other participant the con arguments in an argumentative dialogue). The roles component is not built in the hierarchical order of play, scene, and scriptlet, but lies crosswise to the other components and even goes beyond one play, as the role is stable over the course of several plays.

In summary, collaboration scripts provide a feasible framework for studying expertise in collaboration for several reasons. From the SToG perspective, the explication and definition of internal collaboration scripts seems useful. Furthermore, the explication of goals as a structural component seems useful since goals can be formulated and therefore have the potential to be assessable. Additionally, collaboration scripts provide the base for a measure of knowledge regarding collaboration, largely independent of the knowledge of the content domain. In this way, it is possible to conduct empirical studies regarding the relation between collaboration scripts and knowledge of the content domain. There are tasks (e.g., in an operating room or an emergency situation) for which a high level of collaboration expertise and a high amount of content knowledge are necessary to successfully find a solution. Then, there are tasks (e.g., deciding alone on a working diagnosis from a case file) for which a rather high amount of content knowledge, but almost no amount of collaborative knowledge, is necessary. Further, there are tasks where a high amount of collaboration expertise but little content knowledge is necessary. Our studies, described below, use the latter task, namely, a memory task, which uses only a little content knowledge but a high amount of collaboration expertise.

### Research Questions

As experts have a better organization of procedural knowledge structures than do novices, an expert in collaboration should employ elaborated procedural knowledge regarding collaborative situations (internal collaboration scripts); novice scripts are not as elaborated. Collaboration experts are those who consistently collaborate and outperform others in collaborative situations. According to the features of expertise outlined previously, collaboration experts should create more effective solutions in collaborative situations, be effective in recognizing the structure underlying collaborative situations, possess extensive memory for collaborative information, and have a more structured knowledge than novices.

Thus far, however, the question of how to empirically and reliably assess the difference between novices’ and experts’ internal collaboration scripts remains unanswered. More specifically, whether experts in collaboration (who have proven knowledgeable via a standardized test in one content domain) can apply their internal collaboration scripts when confronted with a collaborative situation of another content domain is an open empirical question. Therefore, the following explorative research questions are to be inquired in two studies:

1. Is there evidence of collaboration expertise?
2. Is there evidence for cross-domain application of collaboration expertise?

### Hypothesis of Study 1

Experts are better able than novices to recall collaboration-specific information after participating in a collaborative situation. In other words, after being confronted with a collaborative situation, collaboration experts should be able to easily recall their internal collaboration scripts, whereas novices do not hold these knowledge aspects.

### Hypothesis of Study 2

If collaboration expertise can be applied across domains, a confrontation with a collaborative situation, regardless of the content of the domain, should activate an expert’s internal collaboration script. Experts will apply this procedural knowledge when asked to recall this situation or prospect how it might continue. Experts would then outperform the novices when comparing the collaboration script information.

If collaboration expertise is not domain-general, a confrontation with a collaborative situation in their content domain should activate an expert’s internal collaboration scripts. Experts will apply this procedural knowledge when asked only to recall this situation or prospect how it might continue. Experts will not apply this procedural knowledge when a collaborative situation outside their content domain is used, but rather recall superficial information, in a manner comparable to that of novices.

## Method

For Study 1 and Study 2 the research was approved by the Ethical Committee of the medical faculty of Ludwig-Maximilians-Universität München (LMU); the original data has been restricted and cannot be made publicly available due to individual-related data. A de-identified dataset can be made available upon request. Quotations used in this article are therefor paraphrased. The individuals seen on the figures in this manuscript have given written informed consent (as outlined in PLOS consent form) to publish these.

### Study 1

Ten collaboration novices (M = 23 years, SD = 2.4 years; six females), who were social-science students from the University of Munich, and ten experts (*M* = 38.40 years, *SD* = 7.32 years; three females) volunteered for the study. To qualify as a collaboration expert, one had to have worked collaboratively during the last seven years for a mean of at least two hours per workday. To control for expertise in the content domain, experts were included in the study only when they had advanced at least one hierarchical level in the social sciences faculty (PhD or specialist degree).

**Fig 1:**
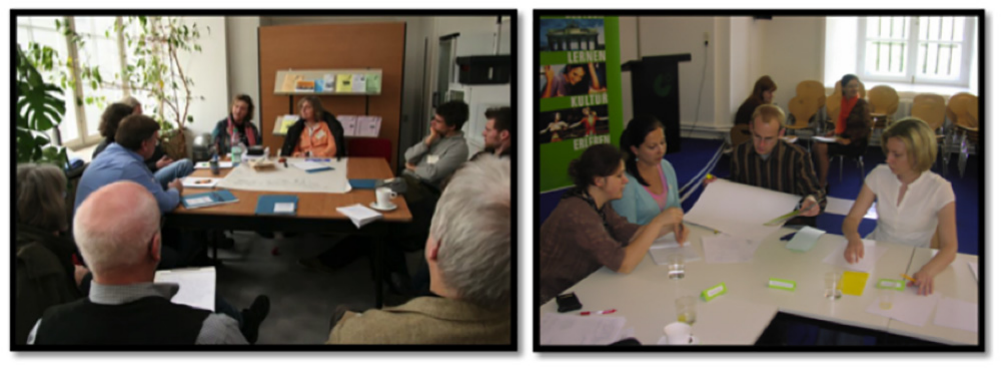
Example of a stimuli used in Study 1.

To trigger collaboration scripts through standardized collaborative situations, novices and experts were shown pictures as stimuli (see Fig 1 and Table 1).

**Table 1:** Description of the stimuli used in Study 1.

Stimulus Number	Content Domain	Type	Description of Stimulus
Stimulus 1	Social Science	Picture	Stimulus 1 is a picture that shows ten people sitting around a table. Clearly five are males and three are females but only the tops of the heads of the other two people are visible, so their genders are indeterminable. On the table are three blue folders and three piles of white paper as well as a large white paper in the center. Two bottles and a cup also are on the table. In the back are two different plants in front of a window.
Stimulus 2	Social Science	Picture	Stimulus 2 is a picture of three females and a male sitting at a corner table in the front and two females sitting on chairs in the back. There are wooden chairs and a conductor’s stand in the back. On the corner table in the front are three glasses, a cup, and four sheets of regular-size paper. The male holds a regular-size and a large piece of paper in his hands.
Stimulus 3	Social Science	Picture	Stimulus 3 is a picture of seven people (four males, three females). Six of them are standing, one female in the back holds a cup in her right hand. There are four blue chairs and a table in the back.
Stimulus 4	Social Science	Picture	Stimulus 4 is a picture of three people (one male on the right and two females) sitting in front of a laptop computer on a table. The female sitting in the middle holds a piece of paper in her hands, which all three are viewing. In front of the other female lies a book on the table.

The pictures represented collaborative situations (workshop or small group work). After a short introduction, the investigator showed the participants one of four pictures for five seconds in counter-balanced order. This method was chosen for two reasons: First, the short-presentation memory recall has already been successfully applied in chess expertise research to distinguish experts’ from novices’ memory structures [13,26,27]. Second, the presentation leads to a task that would confront participants with a collaborative situation and make experts and novices employ their collaboration script without their performance interfering with the collaborative situation itself. After the presentation of the picture, each participant was asked to write down the answer to the “recall question”: *What did you see in the picture*? The same procedure was repeated for another picture. For the remaining pictures the participants were asked to answer three “script questions”: *What has most likely led to the situation*? *What happens in the depicted situation*? *What is most likely to happen next in the situation*? The script questions were used to stimulate answers that include procedural knowledge regarding the depicted situations. Note that all of the questions refer to either the recall of a situation, the speculation of actions that happened, or the prediction of what will happen. No direct questions regarding the employment of collaborative procedural knowledge are asked, and no information regarding the context or hints as to what the picture’s description is used for are given. Therefore, a greater experience in collaborative situations can have developed their internal collaboration scripts and shaped the answers.

The participants were given as much time as they needed to answer. The recall question was used to determine what could be remembered freely; the script questions were chosen to see whether triggering procedural knowledge might make the task easier, to facilitate that the novices employ their internal collaboration script.

### Coding scheme, Study 1 and Study 2

The written data of both studies was analyzed using the same coding scheme, despite that study 1 took place in the domain of the social sciences and study 2 in the domain of medicine.

Two main categories were defined: superficial and script information. To assess detailed collaboration script information, we used the categories of Kollar, Fischer, and Hesse [28]: *goals*, *activities*, *sequences of activities*, and *roles*. Further, we coded superficial information as that which describes information clearly visible in the picture and does not refer to procedural knowledge.

Paraphrased examples of experts and novices from both studies, each for the same stimuli are presented in table 2. Note that medical content knowledge is not coded neither for the experts, nor for the novices.

**Table 2:** Paraphrased Examples of Experts and Novices from Social Sciences and Medicine

Content domain (Group)	Paraphrased Example	Coding	
Social Sciences (Novice)	“A teacher who wears a red sweater stands in front of the classroom and asks the students a question. There are four chairs and two windows in the background.”Nov. 6, Stim. 3	1 activity	ask a question
		2 roles	teacher, students
		0 sequences	
		0 goals	
		3 superficial information	red sweater, four chairs, two windows
Social Sciences (Expert)	“A typical workshop situation, where the participants are in the beginning of refining the expectations of the group towards the workshop. The tutor of the workshop samples the expectations and then summarizes them on the whiteboard in order to structure the day. I would guess the workshop is an annual retreat of a social organization, the external tutor has been invited to.” Exp. 2, Stim. 3	3 activities	refine, sample, summarize
		2 roles	tutor, participant
		1 sequence	then
		1 goal	structure day
		1 superficial information	whiteboard
Medicine (Novice)	“Four people, one of them a nurse and three others stand in front of a bed of a patient and talk to her.” Nov. 4, Stim. 4	1 activity	talk
		1 role	nurse
		0 sequences	
		0 goals	
		0 superficial information	
Medicine (Expert)	“[…] An important point has been raised (by whom is unclear), then they discuss it and the black-haired physician seems to contradict, while the nurse might be asked for her opinion later. They have not found a decision on a treatment for the patient, yet.” Exp. 3, Stim. 4	5 activities	raise, discuss, contradict, ask, decide
		2 roles	nurse, physician
		3 sequences	then, later, yet
		1 goal	find treatment
		1 superficial information	black-haired
Medicine (Novice)	“two physicians have a patient, where the diagnosis is not clear. The physicians need to find the diagnosis, but perhaps other tests like liquor-punction need to be performed.” Nov. 10, Stim. 2	0 activities	
		1 role	physician
		0 sequences	
		1 goal	find diagnosis
		0 superficial information	
Medicine (Expert)	"Two radiologic specialists during interventional diagnosis of the cranium in collaborative consultation. Diagnosis could be found after additional radiologic imaging. But then, most likely more than one specialty will be involved and after discussion a therapeutic decision in this, most-likely oncological, case will be reached.”Exp. 18, Stim. 2	2 activities	consult, discuss
		1 role	specialist
		3 sequences	after, then, after
		2 goals	therapeutic decision, find diagnosis
		0 superficial information	

For Study 1, a student assistant coded all of the transcripts and 10% of the transcripts were also coded by another student assistant (Cohen’s κ_Script information Study1_ = .87; Cohen’s κ_Superficial information Study1_ = .66; Cohen’s κ_Recall Study1_ = .87). The measurement for each independent variable was reliable (Cronbach’s α_Script Information_ = .86; Cronbach’s α_RECALL_ = .74; Cronbachs α_Superficial Information_ = .61).

For Study 2, a student assistant coded all of the transcripts and 10% of the transcripts were recoded (Cohen’s κ_Script information Med Study2_ = .82; Cohen’s κ_Script information Social Science Study2_ = .83; Cohen’s κ_Superficial information Med Study 2_ = .69; Cohen’s κ_Superficial information Social Science Study2_ = .84). The internal consistency of each dependent variable of the measurement is sufficiently high for the medical content domain (Cronbach’s α_Script Information_ = .87; Cronbach’s α_Superficial Information_ = .79; Cronbach’s α_RECALL_ = .64). For the content domain social sciences, Cronbach’s α indicates sufficient reliability for the dependent variable Script information and RECALL, but not for the superficial information (Cronbach’s α_Script Information_. = .59; Cronbach’s α_Superficial Information_ = .25; Cronbach’s α_RECALL_ = .61). Therefore the results regarding superficial information will not be interpreted.

### Data analysis

All data were processed in SPSS 20.0 with an alpha-level of p < .05. When multiple post hoc comparisons with the same data were carried out, the alpha-level was Bonferoni-adjusted [29].

## Results, Study 1

As hypothesized, the collaboration experts recalled more script information than the collaboration novices. Also, more superficial information is recalled by the collaboration experts. For the stimuli in which the recall question was asked, the experts also outperformed the novices with regard to script information (variable: RECALL). For an overview of the descriptive data see Table 3. A t-test revealed that the overall difference of script information between collaboration experts (*M*_experts_ = 25.20, *SD* = 5.88) and collaboration novices (*M*_novices_ = 13.80, *SD* = 4.47) was significant: *t*(18) = 4.88, *p*<0.01, *d* = 2,13. For the variable RECALL, a t-test revealed that collaboration experts (*M*_experts_ = 5.80, *SD* = 1.62) stated significantly more script information than novices (*M*_novices_ = 2.40, *SD* = 2.27): *t*(18) = 3.86, *p*<0.01, *d* = 1.73. This is supporting the hypothesis of study 1.

**Table 3:** Descriptive Statistics of Study 1. Scores represent the number of the information type stated over all the stimuli for both conditions. RECALL is the number of script information deployed after recall questions.

	Collaboration Experts		Collaboration Novices	
	*M*	*SD*	*M*	*SD*
**Script Information**	25.20	5.88	13.80	4.47
** RECALL**	5.80	1.62	2.40	2.27
**Superficial Information**	54.80	23.38	34.10	9.79

## Methods, Study 2

**Fig 2:**
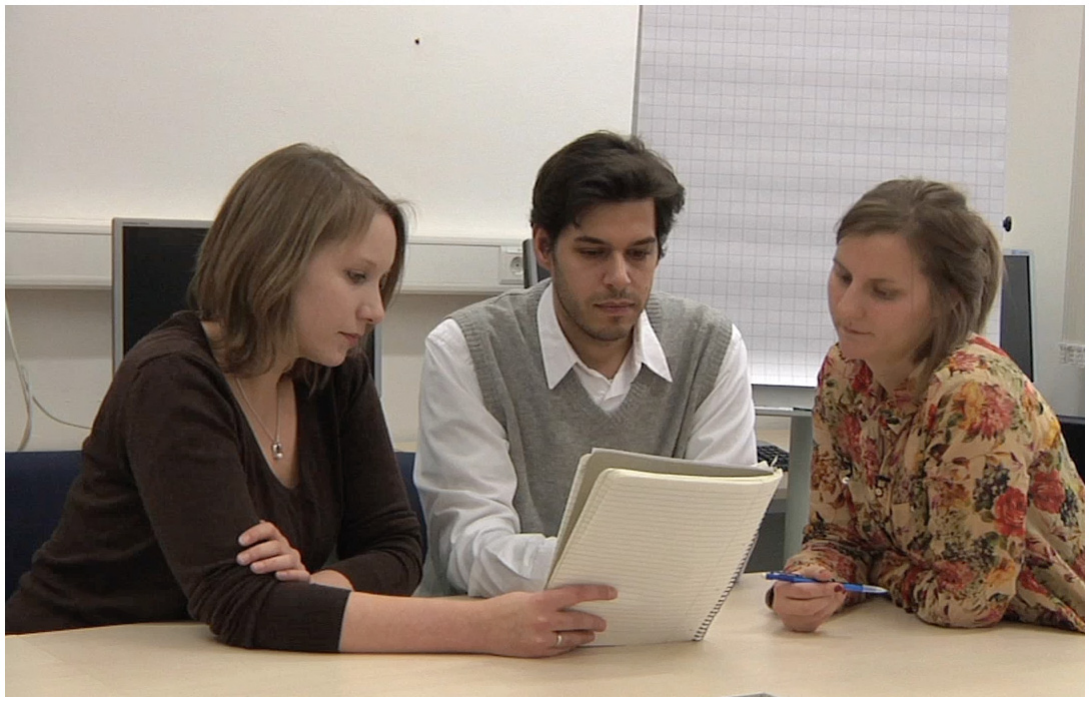
Example of stimuli picture used in Study 2.

Twenty collaboration novices (*M* = 25.75 years, *SD* = 4.7 years; 13 females) and twenty collaboration experts (*M* = 41.57 years, *SD* = 7.87 years; eight females) volunteered for the study. All novices were final year medical students at LMU Munich. Experts were physicians who were specialists needing a high amount of collaboration in their professional lives (internal medicine and anesthesiologists) [30]. Furthermore, all participants completed a questionnaire in which, besides age and sex, they were asked for the number of hours of collaborative work per day. All experts stated that they collaboratively work between 4 and 8 hours per workday. All novices stated that they work collaboratively less than 1 hour per workday. The instruction was reduced to a minimum and no practice trials took place not to bias the participants as to what information would be coded (“You will be shown several pictures and short audio-free videos for 5 seconds in the center of the screen. Afterwards you will be asked questions. Please answer them in the text-boxes provided. You have unlimited time to answer the questions. Do you have any questions? If you are ready for the first picture click the ‘Continue’ button or press ‘Enter’”). One of eight stimuli (four videos with no audio, four pictures) containing collaborative situations in medical contexts was shown to each participant for five seconds (for an example, see Fig 2).

The same procedure was repeated eight times; four times asking a recall question and four times asking script questions, controlling for a balanced combination of stimuli type (picture/video) and question type (recall/script questions) as described above. After the eighth stimulus, the participants were shown two collaborative situations of a social science setting to compare their answers to the answers from their own content domain. The order of questions was balanced over all participants for the social science setting as well.

Note that we asked for a prediction of a situation unfamiliar to the participants. The employment of a script from the procedural memory we coded as a collaboration script. A variety of collaborative situations from the content domain was depicted. We assumed that the experts had definitely been in all the situations from their content domain for several times and therefore were likely to having built up internal collaboration scripts. For better comparability two stimuli were included in both content domains, where, besides superficial objects and clothing, an almost similar stimulus from the other content domain was included. For example, other than the clothing and a notepad instead of a patient chart, stimuli 5 and 10 are almost identical but superficially (patient chart in medical content domain and memo pad in the social science content domain) look quite different. Descriptions of each of the stimuli are given in Table 4. Over the course of the study, the order in which the stimuli were presented was counter-balanced. Answering time was not limited, but was registered. The analysis of answering time as a an indicator of internalization was analyzed and reported elsewhere [31].

**Table 4:** Description of the stimuli used in Study 2. The stimuli which are similar with respect to collaboration, but differ in content domain and superficial attributes are marked accordingly.

Stimulus Number	Content Domain	Type	Description of Stimulus
Stimulus 1	Medical	Picture	Stimulus 1 contains a picture of a surgical theatre in which one surgeon wearing surgical loupes performs the operation, two assistant nurses help, and three persons watch the operation. All wear surgical masks and scrubs.
Stimulus 2	Medical	Picture	Stimulus 2 shows one male and one female physician who collaboratively study an MRI picture of a patient’s head.
Stimulus 3 Medical	Medical	Picture	Stimulus 3 shows three physicians (one female, two males) and one nurse in a patient’s room (the female patient is visible) performing a ward-round, as all physicians look at a patient chart that the female physician holds in her hands.
Stimulus 4 Medical	Medical	Picture	Stimulus 4 contains a ward-round in an intensive care setting. Two male physicians stand on the far side of the patient bed, one male physician presumably with the patient chart in one hand on the near side of the patient.
Stimulus 5 (similar to stimulus 10 with respect to collaboration)	Medical	Video	Stimulus 5 shows a short sequence in a patient room, where three physicians stand together and one physician supposedly makes a suggestion while the other two nod.
Stimulus 6	Medical	Video	Stimulus 6 shows three physicians and one nurse in a patient room. In the short sequence, one physician looks from the patient to another physician three times.
Stimulus 7	Medical	Video	Stimulus 7 shows a short sequence of one male and two female physicians. The female physician in the middle holds the patient chart and the male doctor talks to her, she talks back one sentence and nods, and the other female physician also looks at the patient chart.
Stimulus 8 (similar to stimulus 9 with respect to collaboration)	Medical	Video	Stimulus 8 is a short video sequence in which two female physicians and one male physician look at a patient chart that the male doctor on the right side holds. The female physician on the left side points at something in the chart and both of the other physicians look from one to the other before the male physician starts nodding.
Stimulus 9 (similar to stimulus 8 with respect to collaboration)	Social Science	Picture	Stimulus 9 is a picture of three people (one male on the right and two females) sitting in front of a laptop computer on a table. The female sitting in the middle holds a piece of paper in her hands, which all three of them view. In front of the other female lies a book on the table.
Stimulus 10 (similar to stimulus 5 with respect to collaboration)	Social Science	Video	Stimulus 10 is a video sequence showing three people (one male, two females) in which the female on the left holds a memo pad out to both of the others. The male points to something on the paper, both of the other persons look at each other, and one of the females starts nodding.

## Results, Study 2

As hypothesized, the collaboration experts stated more script information (*M*_experts_ = 71.65, *SD* = 33.23) than the novices (*M*_novices_ = 54.25, *SD* = 15.01) in the medical domain. A MANOVA revealed that this difference was significant with a medium-sized effect, *F*(1;38) = 4.16; η^2^ = .10. Furthermore, when stimulated by videos (variable: VIDEO), the experts (*M*_experts_ = 36.70, *SD* = 16.77) stated more script information than the novices (*M*_novices_ = 27.95, *SD* = 8.74). The calculated MANOVA revealed this difference as significant with a medium-sized effect, *F*(1;38) = 4.55; η^2^ = .11. For the pictures (variable: PICTURE), a similar difference was found (*M*_experts_ = 34.95, *SD* = 17.48; *M*_novices_ = 26.30, *SD* = 7.02) with a medium-sized effect as well (*F*(1;38) = 4.28; η^2^ = .10). Outside the medical domain, neither the overall difference (*M*_experts_ = 11.70, *SD* = 6.79; M_novices_ = 9.65, *SD* = 4.17, p = .26) nor the difference for the video (*M*_experts_ = 5.10, *SD* = 4.60; *M*_novices_ = 5.00, *SD* = 4.14, p = .09) or the picture (*M*_experts_ = 6.60, *SD* = 4.44; *M*_novices_ = 4.65, *SD* = 2.39, p = .93) was significant. In the medical content domain, the experts did not differ from the novices regarding the superficial information; for social sciences the measurement consistency was too low to be reasonably interpreted. All descriptive data of Study 2 is summarized in Table 5.

**Table 5:** Descriptive Statistics of Study 2. Scores represent the number of the information type stated over all the stimuli by the subsequent group. RECALL is the number of script information answered only on the recall questions.

	Collaboration Experts		Collaboration Novices		MANOVA (Experts-Novices)	
	*M*	*SD*	*M*	*SD*	*F*	η^2^
**Medical content domain**						
** Superficial information**	23.65	20.78	26.10	10.62	n.s.	
** Script information**	71.65	33.23	54.25	15.01	4.16	.10
VIDEO	36.70	16.77	27.95	8.74	4.55	.11
PICTURE	34.95	17.48	26.30	7.02	4.28	.10
RECALL	22.65	12.89	16.10	5.15	4.36	.10
**Social science content domain**						
** Superficial information**	7.25	6.31	9.20	5.40	unreliable	
** Script information**	11.70	6.79	9.65	4.17	n.s.	
VIDEO	5.10	4.60	5.00	4.14	n.s.	
PICTURE	6.60	4.44	4.65	2.39	n.s.	
RECALL	3.25	2.96	2.60	1.23	n.s.	

## Discussion

Experts differ from novices consistently regarding their knowledge of collaborative situations. The collaboration experts were able to draw on knowledge from their internal scripts, regardless of the question they are asked, as long as the question remains in the experts’ own content domain. The difference between collaboration experts and novices of retrieved and stated script information is not significant when confronted with situations outside their content domains. Our findings can be taken as evidence that the knowledge on collaboration does not easily transfer to an unfamiliar content domain and thus rather indicates that cross-domain application is unlikely.

### Expertise in collaboration

The results indicate that collaboration can indeed be viewed as a domain of expertise. In general, the differences between novices and experts are underestimated [32] because the tasks performable by experts as well as novices are typically too easy for experts. Either way, the differences between experts and novices were medium-sized to very large [29]. Experts seem to deploy their collaboration scripts to any given situation of their content domain, whereas this did not appear to happen easily in other content domains. It seems that expertise in collaboration, as well as other expert performance, is limited in its scope, and expert performance does not transfer to other domains [4]. Comparably for another domain of expertise, clinical reasoning, i.e. physicians’ think and decision processes to find out a patients’ diagnosis, it was shown that, content-specific illness scripts largely determine diagnostic performance [33]. While there are also examples that some domain-independent structure might generally underlie all diagnostic problems [34]; a direct relationship of this metacognitive knowledge and performance is missing.

### Collaboration scripts

Methodologically, the studies presented here are a first attempt to empirically assess the difference between novices’ and experts’ internal collaboration scripts. We now discuss assessment, internalization, and transfer of collaboration scripts.

We have developed a coding scheme based on a theoretical model to assess collaboration scripts. The sizes of the differences between collaboration experts and novices indicate that the conceptualization of internal collaboration scripts as sketched recently in the SToG [17] seems to be working well in empirical studies, since the structure of internal collaboration scripts is assessable through their goals, activities, sequences of activities, and roles, with a sufficient inter-rater reliability. When developed further, the approach presented here could be used to assess collaboration expertise in practice.

A key angle in the use of collaboration scripts has been its theoretical assumption that they are internalized over time [15]. This internalization is important, enabling learners to reapply their internal collaboration scripts in other (unguided) collaborative learning contexts. The results of an empirical study support internalization [35], yet our study raises the questions of whether and how internal collaboration scripts can be reapplied in other (learning) contexts. Seidel and Stürmer [18] recently developed a video-based instrument for professional vision for pre-service teachers. Professional vision in the classroom means that a teacher is able to make use of professional knowledge when interpreting classroom situations. It was found that professional vision can be measured by using video clips prompting questions to describe, explain, and predict classroom situations. This relates to collaboration scripts, as the questions focus on procedural knowledge. It would be interesting to see studies that as well research internal collaboration scripts of teachers.

In Study 2, no evidence was found that internal collaboration scripts are applicable in other contexts, thus indicating no evidence for cross-domain application. The main difference between the study supporting internalization and reapplication [35] and our study might be that the transfer distance between collaboration in the two content domains in our study might have been higher. To further investigate domain specificity of collaboration expertise, we need studies that vary the similarity/dissimilarity of the content domains with respect to the characteristics of the collaborative situations.

### Limitations

The studies reported here were conducted with a small number of participants because large effect sizes were expected based on prior expertise studies. The effects actually appeared with the estimated magnitude for medical stimuli. We hence argue that the sample size, although not large, is appropriate under the given circumstances. With respect to the social science domain the sample was definitely too small to reliably test the effects. However, effects of this small size (less than one third of a standard deviation) can hardly be considered as expertise effects and their practical importance thus arguable.

In Study 2, due to limited test time, it was not possible to use as many collaborative situations for the other content domain (two stimuli) as the ones that were used for their own content domain (eight stimuli). This might have affected the results regarding the content domain dependence of collaboration expertise, as differences might have shown up with a more balanced plan. Still, the results are based on analyses with the same coding scheme and the impairment was the same for both experts and novices. The internal consistency for the script information was high enough so that differences could have shown. Further the two stimuli were chosen with great care; i.e. stimulus 5 and stimulus 10 are identical, beside hospital clothes and a patient chart. However in the social science content domain of stimulus 10 the experts were not able to deploy script information, while in stimulus 2 they could.

A further critical point is that collaboration expertise was determined with self-report measures a priori. Surely, self-report measures of collaboration expertise are not optimal. Consequently, performance measures in Study 2 revealed large standard deviations, implying that within the group of both experts and novices, there was heterogeneity regarding collaboration expertise. However, the stable result patterns of Study 1 and Study 2 extenuates this limitation somewhat as the effect sizes of the comparisons between experts and novices are expectedly large.

Finally, domain and collaboration expertise are to some extent deliberately confounded in our studies. In Study 1 and Study 2 we sampled professionals with a high amount of content-specific *as well as* collaboration knowledge and compared them to students with a low amount of knowledge in both aspects. However, when studying collaboration in professional fields it is problematic to completely eliminate a confoundation of these variables. For this study, we decided to employ a task for which domain expertise plays only a small role. This has the advantage that even novices could have used collaborative knowledge that they might have developed from other fields. We coded only collaboration scripts independent of the content knowledge to reduce the influence on our results to a minimum. However, based on this study one can not ultimately decide whether cross-domain application in more domain-specific tasks would be possible.

In future studies, the level of confoundation within the sample could be lowered, (i.e., a control group of physicians who don’t need a high degree of collaboration within their professional life, but already have their specialist degree), which would strengthen the assessment and conclusions drawn here. As the effect sizes might be lower in such a comparison, a larger sample sizes might be needed.

Our study focused on only part of the attributes of expertise mentioned in the beginning of the article. We did not get into further detail regarding the structure of the scripts. A comparable qualitative study focusing on the single scenes and scriptlets would help to gain insight into experts’ minds regarding specific collaboration scripts. Further, a relationship of answering time and recalled script information could help to make inferences on internalization of scriptlets. Retrieving collaboration information from memory is not actual collaborative action, so our claims would also be strengthened by use of behavioral measures.

### Conclusions

At this point, we return to the example described at the beginning of the article. In the hospital, the chief of medicine was able to deploy his expert content knowledge in medicine together with his expertise in collaboration. It seems as if he did not even have to consciously think about what to do. Through the repetition of the patient’s symptoms he was able to activate his illness script. Having a fairly good idea about what was wrong with the patient, he applied his elaborated procedural knowledge regarding collaborative situations, or internal collaboration scripts, and he asked questions, thus stimulating collaborative activity in his colleagues and fostering a collaborative trajectory toward a diagnosis. It seems unlikely however, that this expert is able to apply much of his *medical* collaboration expertise to other contexts. So it seems likely that this chief of medicine would act like a novice in the apartment owners meeting.
